# Supplementary material for: Identification of Freezing-Responsive microRNAs and Their Targets in Chinese Jujube by Small RNA and Degradome Sequencing
Source: Int J Mol Sci. 2026 Sep 4;27(17):7912. doi: 10.3390/ijms27177912 (PMC13566890; doi:10.3390/ijms27177912)
Supplement: Supplementary file 1 [file ijms-27-07912-s001.zip › Supplementary Material S2/novel_miR_66.pdf]

Provisional ID : novel_miR_66
Score total : 1.6
Score for star read(s) : -1.3
Score for read counts : 0
Score for mfe : 1.3
Score for randfold : 1.6
Score for cons. seed :
Total read count : 55
Mature read count : 54
Loop read count : 1
Star read count : 0

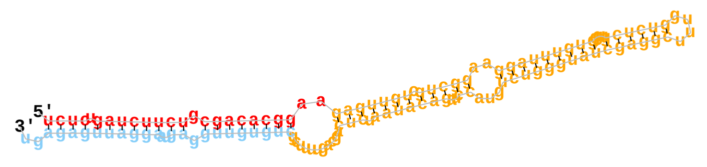

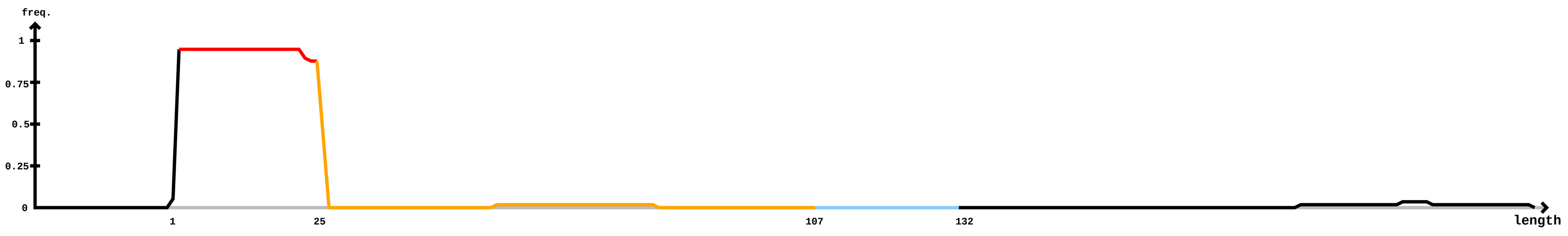


```
                                          Mature                                                                                                    Star
5'- uagauuucucagguaaggagauucucugaucuucugcgacacggaagaguugucgucggaaggauuuguggguugcucugguuucggagcuaugggucuguaccuagacauaaucucagcaguuucgcuguguuggaguaaggauugagagucuugaucaugguuggaauuuggugcuaauuugcuaacuuauuuucuucacuccgguuaggaagauaggagagcaauggaggcaagaacuauuuugcg  -3'  exp
    (((((((((((((...(((((((((.((((((((.((((((((..(((((((.(((((..(((((((((....((((((....))))))))))))))))...))..))))))).)))..........)))))))).))..)))))))))))))))).))).)))..))))))))...((...(((((..(((((((((((.((....)).)))))))))))..)))))...))((((((....)))))).   reads  mm  sample
    .....................uucucugaucuucugcgacacC..............................................................................................................................................................................................................   3      1   LCK-3
    ......................ucucugaucuucugcgacacgg.............................................................................................................................................................................................................   1      0   LT-2
    ......................ucucugaucuucugcgacacggaa...........................................................................................................................................................................................................   27     0   LCK-2
    ......................ucucugaucuucugcgacacggaa...........................................................................................................................................................................................................   23     0   LCK-1
    ...........................................................................cucugguuucggagcuaugggucugua...................................................................................................................................................   1      0   LT-3
    ................................................................................................................................................................................................................uaggaagauaggagagcaaugg...................   1      0   LT-3
    .................................................................................................................................................................................................................................aauggaggcaagaacuauuuug..   1      0   LT-1
```
